# Abnormal sensorimotor cortex and thalamo-cortical networks in familial adult myoclonic epilepsy type 2: pathophysiology and diagnostic implications

**DOI:** 10.1093/braincomms/fcac037

**Published:** 2022-02-15

**Authors:** Raffaele Dubbioso, Pasquale Striano, Leo Tomasevic, Leonilda Bilo, Marcello Esposito, Fiore Manganelli, Antonietta Coppola

**Affiliations:** 1 Department of Neuroscience, Odontostomatology and Reproductive Sciences, Federico II University, Naples, Italy; 2 Department of Neurosciences, Rehabilitation, Ophthalmology, Genetics, Maternal and Child Health (DiNOGMI), University of Genoa, Genoa, Italy; 3 IRCCS Istituto Giannina Gaslini, Genoa, Italy; 4 Danish Research Centre for Magnetic Resonance (DRCMR), Copenhagen University, Kobenhavn, Denmark; 5 Neurophysiology Unit, AORN Cardarelli, Naples, Italy

**Keywords:** TMS, EEG, somatosensory evoked potential, cortical tremor, myoclonus

## Abstract

Familial adult myoclonic epilepsy type 2 is a hereditary condition characterized by cortical tremor, myoclonus and epilepsy. It belongs to the spectrum of cortical myoclonus and the sensorimotor cortex hyperexcitability represents an important pathogenic mechanism underlying this condition. Besides pericentral cortical structures, the impairment of subcortical networks seems also to play a pathogenetic role, mainly via the thalamo-cortical pathway. However, the mechanisms underlying cortical–subcortical circuits dysfunction, as well as their impact on clinical manifestations, are still unknown. Therefore, the main aims of our study were to systematically study with an extensive electrophysiological battery, the cortical sensorimotor, as well as thalamo-cortical networks in genetically confirmed familial adult myoclonic epilepsy patients and to establish reliable neurophysiological biomarkers for the diagnosis.

In 26 familial myoclonic epilepsy subjects, harbouring the intronic ATTTC repeat expansion in the *StAR-related lipid transfer domain-containing 7* gene, 17 juvenile myoclonic epilepsy patients and 22 healthy controls, we evaluated the facilitatory and inhibitory circuits within the primary motor cortex using single and paired-pulse transcranial magnetic stimulation paradigms. We also probed the excitability of the somatosensory, as well as the thalamo-somatosensory cortex connection by using *ad hoc* somatosensory evoked potential protocols. The sensitivity and specificity of transcranial magnetic stimulation and somatosensory evoked potential metrics were derived from receiver operating curve analysis. Familial adult myoclonic epilepsy patients displayed increased facilitation and decreased inhibition within the sensorimotor cortex compared with juvenile myoclonic epilepsy patients (all *P* < 0.05) and healthy controls (all *P* < 0.05). Somatosensory evoked potential protocols also displayed a significant reduction of early high-frequency oscillations and less inhibition at paired-pulse protocol, suggesting a concomitant failure of thalamo-somatosensory cortex circuits. Disease onset and duration and myoclonus severity did not correlate either with sensorimotor hyperexcitability or thalamo-cortical measures (all *P* > 0.05). Patients with a longer disease duration had more severe myoclonus (*r* = 0.467, *P* = 0.02) associated with a lower frequency (*r* = −0.607, *P* = 0.001) and higher power of tremor (*r* = 0.479, *P* = 0.02). Finally, familial adult myoclonic epilepsy was reliably diagnosed using transcranial magnetic stimulation, demonstrating its superiority as a diagnostic factor compared to somatosensory evoked potential measures. In conclusion, deficits of sensorimotor cortical and thalamo-cortical circuits are involved in the pathophysiology of familial adult myoclonic epilepsy even if these alterations are not associated with clinical severity. Transcranial magnetic stimulation-based measurements display an overall higher accuracy than somatosensory evoked potential parameters to reliably distinguish familial adult myoclonic epilepsy from juvenile myoclonic epilepsy and healthy controls.

## Teaser

Dubbioso *et al*. report that sensory–motor cortical hyperexcitability, as well as thalamo-cortical disconnection are involved in familial adult myoclonic epilepsy type 2 pathophysiology. Transcranial magnetic stimulation-based measurements display a higher diagnostic accuracy than somatosensory evoked potential parameters to reliably distinguish familial adult myoclonic epilepsy type 2 from juvenile myoclonic epilepsy and healthy controls.

## Graphical Abstract

**Graphical Abstract:**
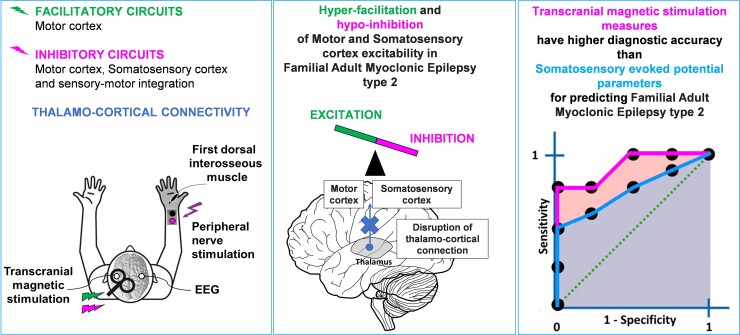

## Introduction

Familial adult myoclonic epilepsy (FAME) also known as familial cortical myoclonic tremor and epilepsy is an autosomal-dominant condition featuring cortical myoclonic tremor, myoclonus and epilepsy.^1–4^

It belongs to the spectrum of cortical myoclonus^5^ and the sensorimotor cortex hyperexcitability represents an important pathogenic mechanism underlying this condition.^6^ The typical findings include a giant somatosensory evoked potential (SEP), the presence of a long loop reflex1, also known as C-reflex, a positive–negative biphasic wave preceding the EMG bursts at the jerk-locked back averaging (JLA).^7,8^

Very few studies on single families, assessing primary motor cortex (M1) excitability using transcranial magnetic stimulation (TMS), showed conflicting results. Some authors demonstrated a direct increase in cortical neuronal excitability by reduced motor thresholds^9,10^ associated with disinhibition of cortical inhibitory circuits, indexed by a reduction of short-interval intracortical inhibition (SICI) and a shortening of the cortical silent period (CSP).^9,10^ However, such results were not entirely confirmed by another independent study performed on a single Dutch family, where motor thresholds were within normal limits and only SICI was found to be altered.^11^

Additional investigations, such as EEG–EMG coherence studies, have further shown the cortical drive of the myoclonus^9,12^ with the strongest coherence observed over the contralateral rolandic area.^9^

Other than pericentral cortical structures, the impairment of subcortical networks seems to play a role in the pathophysiology of FAME. Indeed neuroimaging^13–15^ and pathological evidence^2,11,16–18^ showed an impaired cerebellar-cortical connection, via the thalamo-cortical pathway. Additionally, a causative intronic ATTTC repeat expansion in the *StAR-related lipid transfer domain-containing 7* (*STARD7*) gene, broadly expressed throughout cortical and subcortical structures, has been associated with FAME2.^1^ Such evidence raises the question that not only the sensorimotor cortex but also subcortical circuits, such as the thalamo-cortical pathway could play an additional role in driving cortical hyperexcitability and disease severity.^6,19^

**Figure 1:**
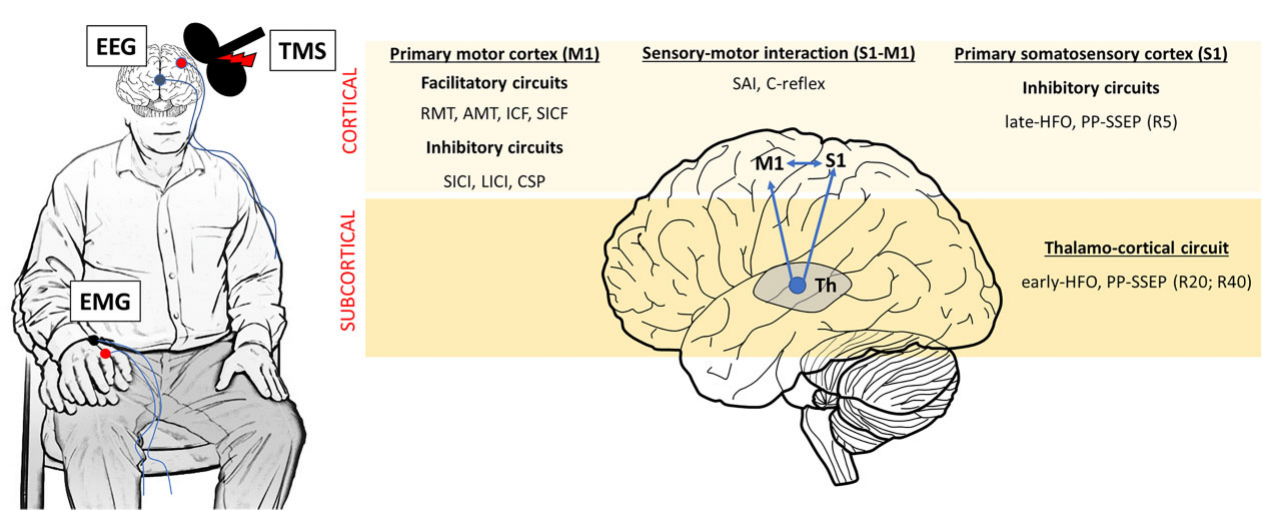
**Overview of neurophysiological protocols to evaluate cortical and subcortical circuits**. During the experiment, patients and healthy controls underwent an extensive neurophysiological battery aimed to evaluate the excitability of cortical circuits within the M1, S1 and their interaction (S1–M1). Specifically, in the M1, we probed facilitatory circuits, such as RMT, AMT, ICF and SICF, inhibitory circuits such as LICI, SICI and CSP. Inhibitory circuits within S1 were evaluated with the late component of the l-HFO and the PP-SEP at ISI of 5 ms (R5). The integration between the two cortices was evaluated using SAI and the cortical reflex (C-reflex). We also evaluated subcortical networks, such as thalamo-cortical circuits with longer ISIs of 20 and 40 ms (R20, R40) at PP-SEP and the e-HFO.

Herein, we employed an extensive neurophysiological battery to track the cortical and subcortical circuits likely underlying sensorimotor hyperexcitability and to get measures associated with clinical severity. Specifically, we used single- and paired-pulse TMS and SEP protocols to evaluate the excitability of the sensorimotor cortex and its connection with subcortical structures, such as the thalamus (Fig. 1 and Table 1).

**Table 1:** Description of neurophysiological protocols assessing cortical and subcortical excitability

Measures	Protocol	Pharmacological manipulation of TMS protocols	Physiological meaning
Primary motor cortex (M1)			
Motor thresholds RMT and AMT	Smallest intensity required to elicit MEPs of ≥50 µV (RMT) or ≥150–200 µV (AMT) in 5 out 10 trials	Increased by voltage-gated sodium channel blockers ( 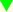 ) Reduced by NMDA-type and AMPA-type glutamate receptor antagonists ( 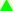 )	Synaptic excitability in M1 Excitability of axons in M1 activated by TMS
MEP 1 mV	Stimulation intensity which produces a 1 mV peak-to-peak MEP amplitude response	N.A.	Global corticospinal excitability
SICI	Paired-pulse TMS: subthreshold CS (95% AMT) ∼2–3 ms before a suprathreshold TS over M1 (TS_MEP1mV_)	Increased by GABA(A) positive allosteric modulators ( 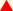 )	Intracortical M1 inhibition
LICI	Paired-pulse TMS: suprathreshold CS (TS_MEP1mV_) 100–150 ms before a suprathreshold TS over M1 (TS_MEP1mV_)	Increased by GABA(B) agonist ( 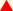 )	Intracortical M1 inhibition
SICF	Paired-pulse TMS: suprathreshold TS over M1(TS_MEP1mV_) 1.0–3.8 ms before subthreshold CS (90% RMT)	Increased by NMDA-type and AMPA-type glutamate receptor antagonists ( 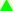 ) Reduced by GABA(A) positive allosteric modulators ( 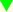 )	Excitability of cortical interneurons and I-waves generation
ICF	Paired-pulse TMS: subthreshold CS (95% AMT) 10–15 ms before a suprathreshold TS over M1 (TS_MEP1mV_)	Reduced by GABA(A) positive allosteric modulators and NMDA-type and AMPA-type glutamate receptor antagonists ( 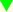 )	Intracortical M1 facilitation
CSP	Suprathreshold TMS (110-130–150% RMT) applied during tonic contraction (∼50% of MVC) of FDI muscle	Increased by GABA(B) agonist ( 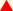 )	Intracortical M1 inhibition
Primary somatosensory cortex (S1) and subcortical connections			
Early HFO	Early component of SSEP high-frequency oscillations	No effects of GABAergic drugs	Activity of thalamo-cortical fibres directed to Brodmann Areas 3B and 1 within S1
Late-HFO	Late component of SSEP high-frequency oscillations	Increased the duration by GABA(A) antagonist	Intracortical inhibition in S1
PP-SEP	Paired-pulse peripheral nerve stimulation: suprathreshold CS 5–20–40 ms before a suprathreshold TS over median nerve (i.e. 120% of motor threshold)	Reduced by GABA(A) agonist ( 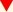 )	Inhibition putatively mediated by local gabaergic inhibitory interneurons (R5) or by cortico-subcortical loops (R20; R40)
S1–M1 connectivity			
SAI	Pairing of a median nerve electrical stimulation 20–22–24–26–28 ms before a suprathreshold TS over M1(TS_MEP1mV_)	Reduced by GABA(A) agonist and Ach antagonist ( 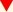 ) Increased by Ach-esterase inhibitors ( 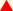 )	Sensory afferent inhibition
C(ortical)-reflex	Late pathological EMG response recorded at ∼40–60 ms after stimulation of the median nerve	N.A.	Electrophysiological correlate of the reflex myoclonic jerk

Ach = acetylcholine; AMPA = α-amino-3-hydroxy-5-methyl-4-isoxazolepropionic acid; AMT = active motor threshold; CS = conditioning stimulus; CSP = cortical silent period; DTC = dentate-thalamo-cortical pathway; GABA = γ-aminobutyric acid; HFO = high-frequency oscillations; ICF = intracortical facilitation; LICI = long-interval intracortical inhibition; MVC = maximum voluntary contraction; MEP = motor evoked potential; N.A. = systematic pharmacological data is not available; NMDA = *N*-methyl-d-aspartate; PP = paired-pulse; RMT = resting motor threshold; SAI = short-latency afferent inhibition; SEP = somatosensory evoked potential; SICF = short-interval intracortical facilitation; SICI = short-interval intracortical inhibition; TMS = transcranial magnetic stimulation; TS = test stimulus; TS_MEP1mV_ = test stimulus adjusted to evoke a MEP of 1 mV amplitude in the right FDI muscle.

The red symbol indicates stronger inhibition (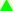) or weaker inhibition (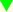), whereas the green symbol indicates stronger facilitation (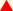) or weaker facilitation (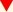); depending on the protocol.

We hypothesized that abnormal cortical excitability is driven by increased facilitation and decreased inhibition within the sensorimotor cortex together with a functional disconnection of thalamo-cortical circuits. Finally, by including juvenile myoclonic epilepsy (JME, Janz syndrome) as a control condition, characterized by cortical sensorimotor hyperexcitability^20–22^ causing myoclonic jerks, we aimed to assess the diagnostic accuracy of TMS and SEP parameters in the differential diagnosis of FAME2 from JME.

## Materials and methods

### Patients

Twenty-six FAME2 patients (8 females, mean age: 45.85 ± 15.65 years), 17 patients with JME (10 females, mean age: 36.29 ± 11.62 years) and 22 healthy controls (HCs) (11 females, mean age: 38.14 ± 14.02 years) were enrolled from January 2017 to January 2019. FAME2 patients presented with autosomal-dominant inheritance, tremulous finger movement, which was increased by action and posture, neurophysiological features consistent with cortical myoclonus (i.e. JLA), and all were confirmed to carry a heterozygous pathogenic intronic (ATTTC)*_n_* insertion in *STARD7* by repeat-primed polymerase chain reaction (PCR) and long-range PCR.^1^ Section 4 of the Unified Myoclonus Rating Scale (UMRS)^23^ was used to assess the severity of cortical myoclonus in FAME2 patients.

Written informed consent was obtained from all participants according to the Declaration of Helsinki. The study protocol was approved by the local ethics committee (University Federico II of Naples, Italy; N. 100/17/ES01).

### Electrophysiologic evaluation

Before starting with TMS protocols, all participants were screened for contraindications to TMS.^24^

During the electrophysiology experiments, subjects were seated comfortably in a reclining chair with the right forearm placed in a prone position on the armrest (Fig. 1).

#### EMG for tremor

Surface EMG recordings from bilateral wrist extensor muscles, wrist flexors and first dorsal interosseous (FDI) were carried out at 256 Hz sampling frequency with Ag–AgCl surface electrodes using a belly-tendon montage (SD Plus amplifier, Micromed, Treviso, IT). Data were monitored using SystemPlus Evolution and stored in a computer for offline analyses. Recordings were performed with arms/wrists outstretched at the shoulder level, which was able to elicit an overt and recordable tremor in all patients. The peak frequency of the tremor and the corresponding peak power were identified with power spectral density estimated on EMG traces (Matlab version 2020b) using the Welch method (500 windows of 4 s with 50% overlap).

#### Measures of inhibition and facilitation within the M1

TMS was delivered with a figure-of-eight coil (MC-B70 with outer diameter of each wing 97 mm) connected to a biphasic MagPro X100 system (MagVenture, Skovlunde, DK) over the left motor cortex to elicit motor evoked potentials (MEPs) at the right FDI muscle.^25^ Resting motor threshold (RMT) and active motor threshold (AMT) were determined according to standard procedures.^26^

Facilitatory circuits were evaluated by the intracortical facilitation (ICF) and the short-interval intracortical facilitation (SICF) protocols, while inhibitory circuits by SICI, long-interval intracortical inhibition (LICI) and CSP.^27^

SICI–ICF, SICF and LICI were studied with a paired-pulse model with a conditioning-test design. For all paradigms, the test stimulus (TS) was adjusted to evoke an MEP of 1 mV amplitude. The interstimulus interval (ISI) between TS and conditioning stimulation differed among protocols (see Supplementary Data and Table 1). Fifteen trials were recorded for each ISI and each protocol randomly intermixed with 15 trials of TS alone (0.2 Hz ± 10%). The ratio of the mean amplitude of the conditioned response to that of the TS response (unconditioned response) was calculated for each condition and ISI in each subject.

#### Measures of primary somatosensory cortex excitability and thalamo-cortical connection

Peripheral nerve stimulation can be used to investigate both primary somatosensory cortex (S1) excitability and thalamo-S1 connection. Specifically, S1 excitability was tested by assessing N20, P25 and N33 amplitudes of SEP, the late component of high-frequency oscillations (l-HFOs) and by the SEPs recovery cycle at the early interval (i.e. 5 ms).^28–30^ Thalamo-cortical connection was assessed by the early component of the HFO (e-HFO)^31^ and late intervals of the SEPs recovery cycle (i.e. 20 and 40 ms),^32,33^ (see Table 1). Several lines of evidence suggest that the l-HFOs are probably located in the primary somatosensory area, specifically Brodmann areas 1 (BA1)^34^ and 3B (BA3B).^35^ The e-HFO represents high-frequency activity from thalamo-cortical fibres projecting mainly to area 3B and 1 within S1.^31^ Regarding SEPs recovery cycle, the suppression of the N20 at short intervals (ISI of 5 ms) is thought to be primarily of cortical origin,^28–30,36^ whereas suppression at longer ISIs (i.e. ISI of 20 and 40 ms) is reported to be mediated by inhibitory postsynaptic interneurons within the dorsal column nuclei and the thalamus (ventral postero-lateral nucleus).^37,38^

SEPs were recorded from CP3 (active) and Fz (reference) according to the international 10–20 system with silver/silver chloride (Ag–AgCl) surface electrodes.

The peripheral stimulation was performed over the right median nerve with the anode placed on the wrist crease and the cathode placed 2 cm proximal. Three thousand monophasic square wave pulses of 200 µs duration were delivered at a frequency of 5 Hz and intensity at 120% of the motor threshold (Digitimer, Welwyn Garden City, UK), which is defined as the minimum stimulation intensity able to produce a small twitch of the abductor pollicis brevis muscle. We chose this frequency of stimulation since a higher stimulus rate (i.e. >10 Hz) significantly decreases the later part of the HFOs after the N20 peak.^31^

One hundred and twenty milliseconds of long EEG trials, starting from 20 ms before the stimulus, were collected at a 5 kHz sampling rate by Signal software and CED 1401 hardware (Cambridge Electronic Design, Cambridge, UK). Pre-processing of the EEG data consisted of cubic-interpolation of the stimulus-related electric artefact from −0.2 to +6 ms^39^ and exclusion of trials with artefacts. N20 peak latency was identified on the average of the trials after band-pass filtering between 1 and 2000 Hz. For HFO 400–800 Hz band-pass filter was used instead and the interval corresponding to e-HFO and l-HFO was identified by peaks exceeding the maximum value of the baseline before and after the N20 latency. The e-HFO and l-HFO areas were calculated separately as areas under the curve corresponding to the two intervals.

To measure the recovery cycle, three supplementary blocks of 750 trials each were recorded while delivering paired pulses at ISI of 5, 20 and 40 ms. From each averaged block of trials, the SEP waveform was subtracted after aligning it to the first stimulus in the paired-pulse waveform. R5, R20 and R40 were defined as the ratio between the response relative to paired-pulse and those relative to single-pulse alone. Amplitude ratios <1 describe a paired-pulse suppression whilst ratios >1 indicate a paired-pulse facilitation.

#### Assessment of sensorimotor (S1–M1) cortical interaction

Sensorimotor integration was evaluated by the short-latency afferent inhibition (SAI)^40,41^ and C-reflex,^42^ (see Table 1). SAI was examined at ISIs ranging from 0 to 8 ms after N20 latency, in steps of 2 ms.^40^ The median nerve was stimulated at the wrist through bipolar surface electrodes (cathode proximal, rectangular pulse of 0.2 ms duration) with an intensity that was adjusted to produce a slight thumb twitch (i.e. 120% peripheral motor threshold). The intensity of the TS was set at an intensity required to elicit a 1 mV MEP in the right FDI muscle. The amplitude of the conditioning MEPs was expressed as a ratio of the mean unconditioned response. Fifteen trials were recorded for each condition and randomly intermixed with 15 trials of TS alone (0.2 Hz ± 10%).

The C-reflex (or long latency reflex I) to electrical stimulation was studied by applying electrical stimulation to the right median nerve at the wrist at the motor threshold and recording EMG activity from bilateral abductor pollicis brevis muscles.^42^ We recorded the latency of ipsilateral and contralateral responses.

### Statistical analysis

Data were analysed using SPSS v. 22.0 (SPSS Inc.) and GraphPad Prism v. 8.4.2 for Windows. Normal distribution was verified using the Kolmogorov and Smirnov tests. Group matching regarding gender and antiseizure medications (ASMs) use were tested using *χ*^2^. One-way ANOVA was applied to compare age, motor thresholds and amplitudes of cortical SEP (N20, P25 and N33) in the three groups: FAME2 patients, JME patients and healthy subjects. Then the effect of different TMS paired-pulse protocols expressed as a percentage of conditioned/unconditioned MEP ratios, and CSP duration were compared with a two-way mixed-model ANOVA, with factor *ISI* for paired-pulse protocols and *stimulation intensity* (110%, 130% and 150% RMT) for CSP as a within-subject factor, and *GROUP* (FAME2 patients, JME patients and HC) as between-subject factor. When dealing with protocols evaluating sensory excitability, a two-way mixed-model ANOVA was performed with factor *ISI* (5, 20 and 40 ms) for the SEP recovery cycle, *component (late versus early)* for HFO as within-subject factor and *GROUP* as the between-subject factor. Furthermore, to rule out any effect of benzodiazepine therapy on neurophysiological metrics in the FAME2 group, we performed a mixed-model ANOVA with *therapy* (FAME2 with versus FAME2 without benzodiazepine therapy) as the between-subject factor and *ISI* (*SICI–ICF*, *SCF*, *LICI*, *SAI*, paired-pulse SEP) or *stimulation intensity (CSP)* or *component (HFO)* as within-subject factor; unpaired *t*-tests were applied for motor thresholds and SEP amplitudes (N20, P25 and N33).

If a significant main effect was obtained in the ANOVA model, group differences were examined with *post hoc* tests (Bonferroni correction for multiple comparisons). The Greenhouse–Geisser method was used to correct for non-sphericity, whenever necessary.

The correlation between clinical variables (disease duration, UMRS score, peak tremor frequency and total power) and the main neurophysiological parameters were evaluated using Pearson’s correlation coefficient. Alpha inflation due to multiple comparisons was controlled according to Bonferroni’s approach when appropriate.

To determine the diagnostic utility of neurophysiological measures to differentiate patients with FAME2 from JME and HC, the area under the curve (AUC), receiver operating characteristic (ROC) curves were used, including a 95% confidence interval (CI) values. Cut-off points were set to minimize the difference between sensitivity and specificity (Youden index). The neurophysiological measures used for ROC analysis were as follows: RMT, AMT, the overall mean of SICI, ICF, SAI, SICF, LICI, CSP, the amplitude of N20, P25 and N33 and the mean area of e-HFO and l-HFO. The *P*-values for AUC were obtained using the statistical test implemented in the GraphPad Prism Software. Specifically, for *P*-values <  0.05, the null hypothesis that the AUC was equal to 0.50 in the population was rejected.

Finally, to compare the ROC curves of TMS measure versus SEP parameters, we performed a pairwise comparison of AUC^43^ derived from TMS metrics and SEP measures to obtain the difference between the areas, the standard error and *P*-value.

Effects were considered significant if *P* < 0.05. All data are presented as mean ± SD (standard deviation) if not stated otherwise.

### Data availability

Patients’ main demographic and clinical data are available in Supplementary Table 1. Other data are available upon reasonable request but cannot be made open because of ethics protocol requirements and the sensitive nature of patient data.

## Results

### Participants

There was no significant difference in age [one-way ANOVA *F*(2,64) = 2.904, *P* = 0.082] and sex (*χ*^2^ = 3.664, *P* = 0.160) among the three groups. Mean disease duration and age of disease onset (tremor or seizure) did not differ (*P* = 0.09 and *P* = 0.21) between FAME2 (25.52 ± 14.33 and 20.85 ± 7.32) and JME patients (17.94 ± 11.92 and 18.35 ± 5.43).

At the evaluation, patients were treated with at least one ASM, except for three FAME2 patients. The percentage of FAME2 patients taking at least one benzodiazepine was significantly higher (*χ*^2^ = 7.904, *P* = 0.005) compared with JME patients. However, among the benzodiazepine treatments, only clonazepam was significantly different between the two groups (*χ*^2^ = 5.467, *P* = 0.019), whilst no significant difference was observed for clobazam (*χ*^2^ = 1.371, *P* = 0.242) and phenobarbital (*χ*^2^ = 0.390, *P* = 0.532).

JME patients, on the other hand, were on lamotrigine therapy in a higher percentage than FAME2 group (*χ*^2^ = 8.925, *P* = 0.003). No significant difference was observed between the two groups for valproate (*χ*^2^ = 1.122, *P* = 0.289), phenytoin treatment (*χ*^2^ = 0.669, *P* = 0.413) and the mean number of ASMs: 2.54 ± 1.9 (FAME2) versus 2.59 ± 1.12 (JME), *P* = 0.91 (Supplementary Table 1 and Fig. 1). Finally, in the FAME2 group, the number of patients on benzodiazepine treatment (*n* = 12) was lower than the number of patients off benzodiazepine therapy (*n* = 15).

### Neurophysiological results

Overall, neurophysiological recordings were well tolerated by both patients and controls, and none of the participants reported side effects.

### Motor thresholds

#### Reduction of RMT, AMT and MEP1mV in FAME2

**Figure 2:**
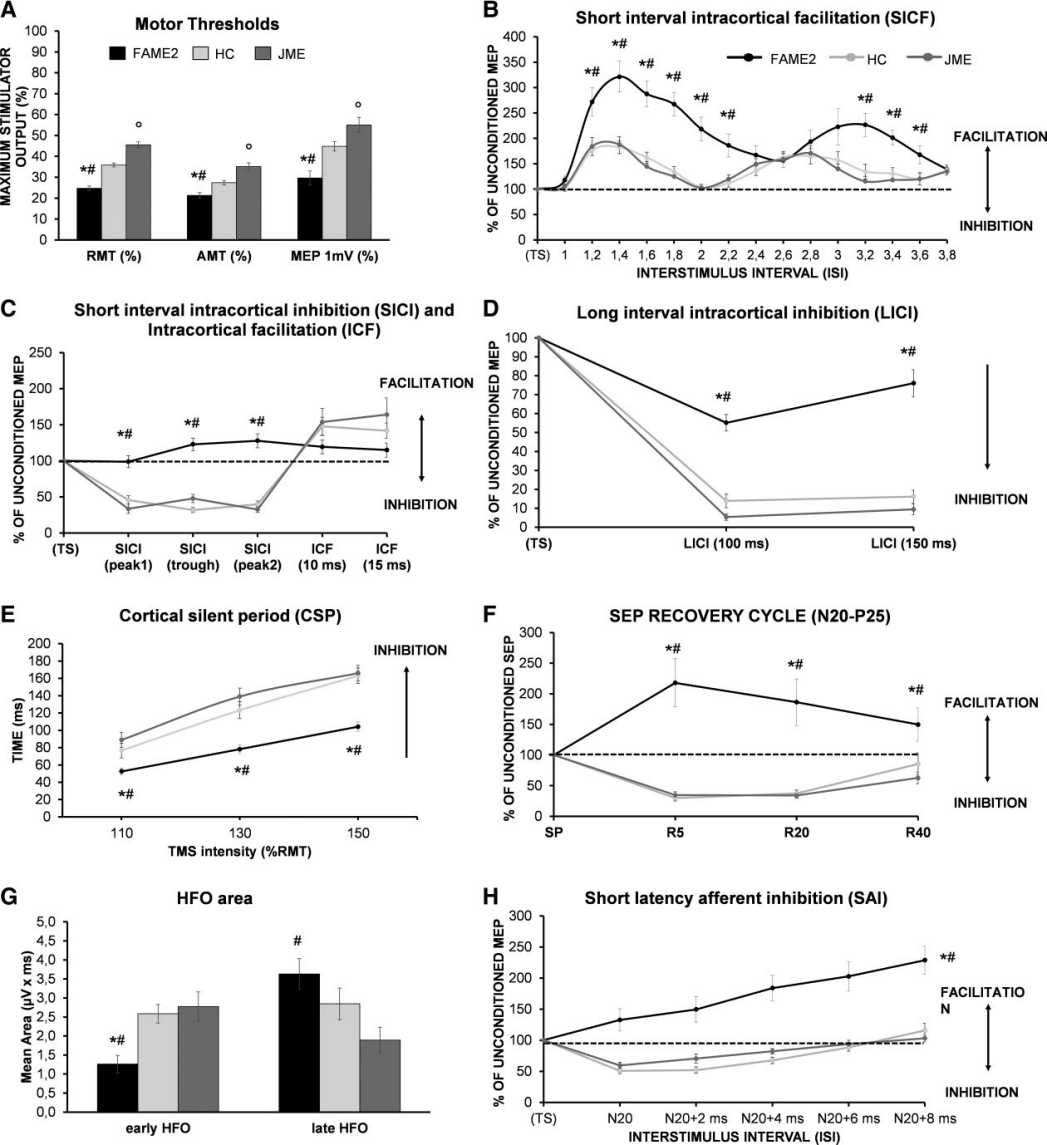
**Assessment of facilitatory and inhibitory circuits in the M1, in the S1 and evaluation of thalamo-cortical connectivity**. Group average of stimulation intensity to produce motor thresholds (RMT, AMT) and 1 mV peak-to-peak MEP amplitude response (MEP 1 mV), showing significant lower values in FAME2 (black bars) compared with HC (light grey bars) and patient with JME (grey bars). JME also displayed significantly higher values with respect to HC (**A**). Group average data are presented as ratio of the conditioned to the unconditioned MEP amplitude for each ISI of SICF, showing significant enhancement of motor cortex facilitatory circuits in FAME2 (black lines) compared with HC (light grey lines) and JME (light grey lines), (**B**). (**C**, **D**, **E** and **H)** show the significant suppression of inhibitory mechanisms probed by SICI, LICI, CSP and SAI in FAME2 compared with JME and HC. Interestingly, in the case of SICI and SAI, the physiological inhibition was replaced by paradoxical facilitation (**C** and **H**). Note that SICI intervals were modelled based on the individual first peak (Peak 1), trough and second peak (Peak 2) obtained from the SICF curve.^44,45^ (**F**) shows the N20 recovery cycle at ISI of 5 (R5), 20 (R20) and 40 ms (R40) demonstrating paradoxical facilitation in FAME2 patients compared with HC and JME patients that exhibit the physiological inhibitory curve. The mean area of the early but not the late component of the high-frequency oscillation (HFO) generated by the conventional SEP showed a significant reduction in FAME2 patients, suggesting an impairment of thalamo-cortical connection (**G**). *HC versus FAME2; **°**HC versus JME; **^#^**FAME2 versus JME. *P*-values computed with one-way ANOVA (**A**) and mixed-model ANOVA (**B–H**). Significant *P* < 0.05. Bar errors indicate standard error of the mean.

One-way ANOVA comparing motor thresholds and the MEP1mV in the three groups showed significant differences for all parameters: RMT [*F*(2,64) = 31.204, *P* < 0.001], AMT [*F*(2,64) = 24.022, *P* < 0.001] and MEP1mV [*F*(2,64) = 33.684, *P* < 0.001]. *Post hoc* analysis showed lower RMT, AMT and MEP1 mV in FAME2 (RMT: 24.73 ± 5.61, AMT: 21.35 ± 4.95, MEP1mV: 29.62 ± 7.07) with respect to JME (RMT: 45.53 ± 13.73, AMT: 35.12 ± 9.52, MEP1mV: 55 ± 15.33) and HC (RMT: 35.86 ± 5.93, AMT: 27.41 ± 4.69, MEP1mV: 44.82 ± 8.3). In addition, JME showed a significantly higher threshold with respect to HC (all *P* < 0.009) (Fig. 2A).

### Facilitatory and inhibitory circuits within M1

Neurophysiological metrics of M1 excitability showed increased facilitation and decreased inhibition in FAME2.

#### Increase of SICF in FAME2

Mixed-model ANOVA revealed increased SICF in FAME2 compared with JME (*P* < 0.001) and HC (*P* < 0.001) with significant effects of *ISI* [*F*(11,638) = 3.541, *P* < 0.001] and *ISI* × *GROUP* interaction [*F*(66,638) = 2.079, *P* < 0.001] (Fig. 2B). *Post hoc* analysis confirmed that SICF at ISIs of 1.2, 1.4, 1.6, 1.8, 2, 2.2, 3.2, 3.4, 3.6 ms was significantly higher in FAME2 compared with the other three groups (all *P* > 0.008). No difference was found between JME and HC (*P* = 0.915). The expected *ISI* effect is due to the temporal dependency of the two facilitatory peaks within MEP responses.

#### SICI decreased, ICF not affected in FAME2

For SICI–ICF, mixed-model ANOVA yielded *a GROUP* [*F*(2,47) = 13.104, *P* < 0.001] and *ISI* × *GROUP* [*F*(8,188) = 13.474, *P* < 0.001] effect, indeed *post hoc* comparisons showed that FAME2 exhibited an overall altered modulation for intracortical circuits, reaching significant effects for SICI protocol (SICI_peak1_, SICI_trough_ and SICI_peak2_, all *P* < 0.001) but not for the ICF protocol (ISI: 10 and 15 ms, all *P* > 0.245) (Fig. 2C). As expected, we also showed the main *ISI* effect [*F*(4,188) = 53.245, *P* < 0.001] because MEPs were inhibited at short ISIs, whereas for longer ISI, the inhibition was replaced by facilitation (Fig. 2C). No significant difference was evident between JME and HC (*P* = 0.248).

#### LICI and CSP decreased in FAME2

**Figure 3:**
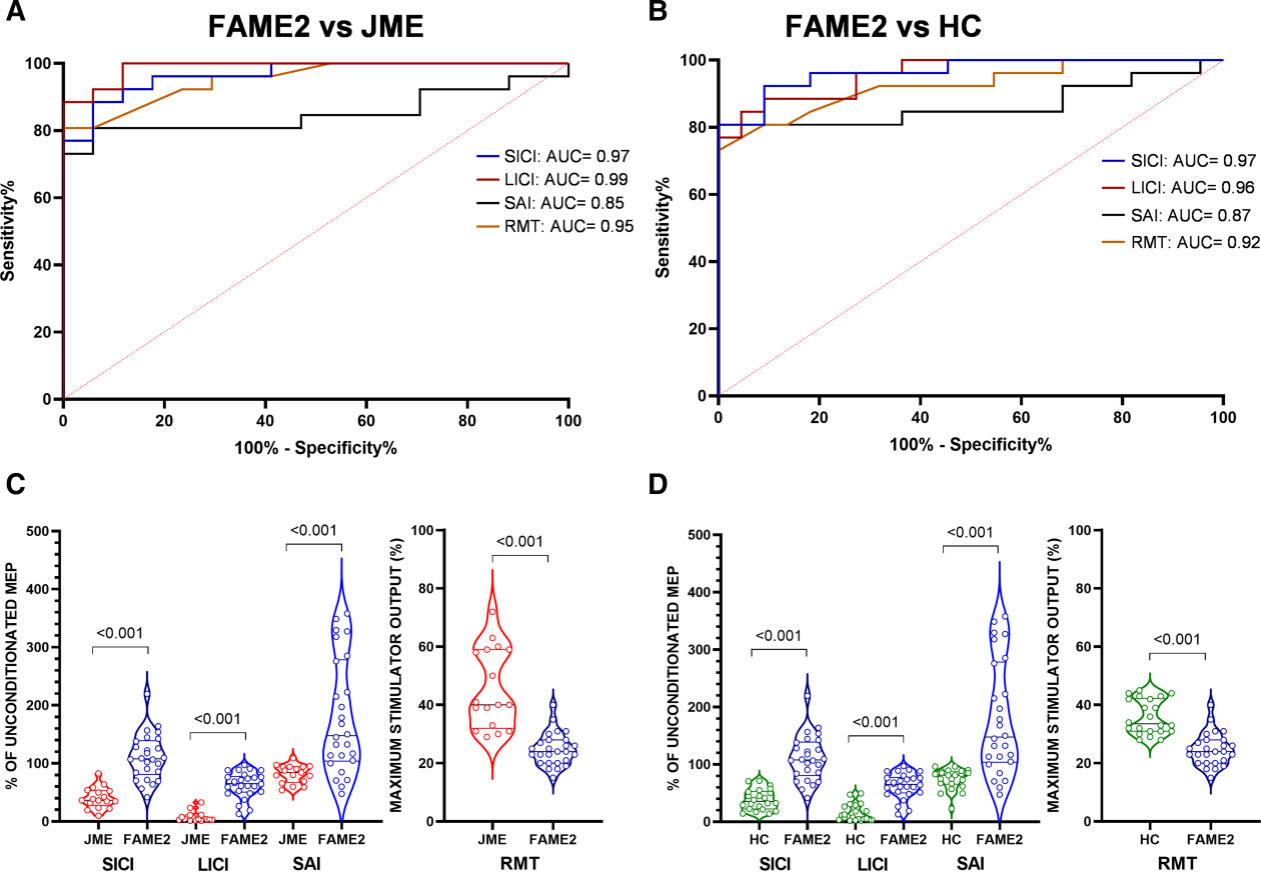
**Diagnostic accuracy of TMS measures**. ROC curve and AUC values for TMS parameters, namely RMT, SICI, LICI and SAI in differentiating patients with FAME2 from those with JME (**A**) and HC (**B**). (**C**) and (**D**) represent the violin plot of the individual data of FAME versus JME (**C**) and FAME2 versus HC (**D**). Data are presented as a ratio of the conditioned to the unconditioned MEP amplitude, except for RMT presented as percentage of maximum stimulator output. RMT = resting motor threshold; SAI = mean short-latency afferent inhibition (0, 2, 4, 6, 8 ms); SICI = mean short-interval intracortical inhibition (ISI_peak1, ISI_trough, ISI_peak2), LICI = mean long-interval intracortical inhibition (100 and 150 ms). *P*-values computed with unpaired *t*-tests (**C** and **D**). Significant *P* < 0.05.

LICI protocol showed a main effect of *GROUP* [*F*(2,62) = 73.468, *P* < 0.001] and *ISI* × *GROUP* [*F*(2,62) = 4.951, *P* = 0.01] effects. Indeed, *post hoc* testing revealed that only FAME2 group exhibited a reduction of the inhibition’s magnitude at each ISI (100 ms: *P* < 0.001, 150 ms: *P* < 0.001) (Fig. 2D). Mixed-model ANOVA also showed the main effect of *ISI* [*F*(1,62) = 12.866, *P* = 0.001], being the overall inhibition at 100 ms stronger than the one obtained at 150 ms for all participants (25.19 ± 27.08 versus 34.98 ± 37.49, *P* = 0.003). Same findings were obtained for CSP, with a main effect of *GROUP* [*F*(2,53) = 20.079, *P* < 0.001] and *stimulation intensity* × *GROUP* interaction [*F*(4,106) = 6.879, *P* < 0.001]. *Post hoc* analysis revealed that FAME2 had a shorter inhibition compared with JME (*P* < 0.001) and HC (*P* < 0.001) at each stimulation intensity (all *P* < 0.008). As expected, the main effect of *stimulation intensity* [*F*(1,28) = 7.851, *P* = 0.009] is due to the well-known CSP length dependency from the intensity of stimulation (Fig. 2E). Contrasting JME versus HC, no significant difference was observed for LICI (*P* = 0.167) and CSP (*P* = 0.251) protocols. Individual data of RMT, SICI and LICI are plotted in Fig. 3C and D.

#### No benzodiazepine effect on M1 protocols in FAME2 patients

To exclude any potential effect of benzodiazepine drugs on intracortical circuits explored by TMS, we compared FAME2 patients with and without benzodiazepine treatment. This analysis did not disclose any effect of benzodiazepine on motor thresholds (unpaired *t*-test: RMT, *P* = 0.65; AMT, *P* = 0.759; MEP1mV, *P* = 0.831), SICI–ICF [*F*(1,13) = 0.377, *P* = 0.55], SICF [*F*(1,5) = 0.108, *P* = 0.756], LICI [*F*(1,17) = 2.082, *P* = 0.167] and CSP [*F*(1,17) = 0.258, *P* = 0.618].

### Somatosensory cortex excitability, S1–M1 integration and thalamo-S1 connection

#### SEP with larger N33 in FAME2

**Figure 4:**
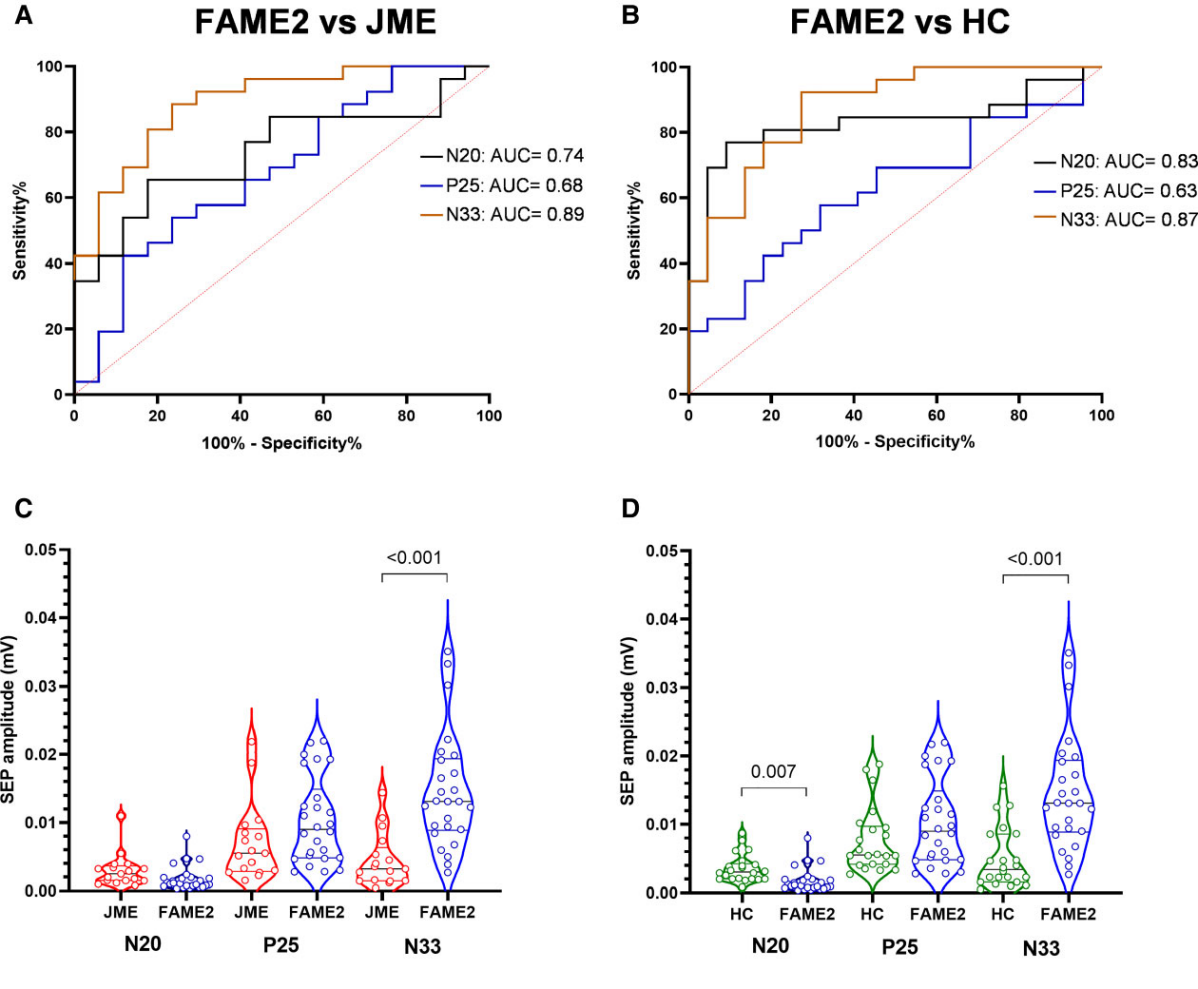
**Diagnostic accuracy of conventional SEP measures**. ROC curve and AUC values for conventional SEP parameters, namely N20, P25 and N33 amplitude, in differentiating patients with FAME2 from those with JME (**A**) and HC (**B**). (**C**) and (**D**) represent the violin plot of the individual data of FAME versus JME (**C**) and FAME2 versus HC (**D**). *P*-values computed with unpaired *t*-tests (**C** and **D**). Significant *P* < 0.05.

Figure 2G and H summarizes the electrophysiological findings related to S1 excitability and sensorimotor integration in patients and HC. Regarding cortical components of SEP, one-way ANOVA showed significant differences only for N20 [*F*(2,60) = 4.223, *P* = 0.019] and N33 [*F*(2,60) = 17.541, *P* < 0.001] but not for P25 [*F*(2,60) = 2.874, *P* = 0.098]. *Post hoc* analysis showed that FAME2 patients had smaller N20 compared with HC (*P* = 0.007) but not to JME (*P* = 0.07) and larger N33 compared with JME (*P* < 0.001) and HC (*P* < 0.001) (see Fig. 4C and D). SEP components did not differ between JME patients and HC (all *P* > 0.419).

#### Paired-pulse SEP inhibition lower at 5, 20 and 40 ms in FAME2

FAME2 displayed a significant reduction of N20-P25 suppression [*GROUP* effect: *F*(2,27) = 23.757, *P* < 0.001, and *ISI* × *GROUP* interaction: *F*(2.834,38.263) = 6.704, *P* < 0.001, Greenhouse–Geisser correction: *ε* = 0.709]. *Post hoc* analysis showed that the impairment of SEP inhibition involved both cortical short interval (5 ms: all *P* < 0.001) and subcortical longer ISIs (20 ms: all *P* < 0.001, 40 ms: all *P* < 0.029) (Fig. 2F). No difference was observed between JME patients and HC (all *P* > 0.570).

#### Reduction of the e-HFO area in FAME2

Analysis of HFO area disclosed a main *component* effect [*F*(1,57) = 4.697, *P* = 0.034], meaning that the e-HFO area was overall smaller than the l-HFO area. In addition, we found a *component* × *GROUP* interaction [*F*(2,57) = 13.260, *P* < 0.001], specifically the e-HFO area in FAME2 patients was significantly smaller than JME (*P* = 0.002) and HC (*P* = 0.003) (Fig. 2G), whilst the l-HFO area of FAME2 was larger than JME (*P* = 0.034) and not HC (*P* = 0.478). JME and HC did not differ for both e-HFO (*P* = 0.676) and l-HFO (*P* = 0.478). Considering the entire population, we observed a positive correlation between the e-HFO and N20 amplitude (*r* = 0.475, *P* < 0.0001) and between l-HFO and N20 (*r* = 0.461, *P* < 0.0001), P25 (*r* = 0.557, *P* < 0.0001) and N33 amplitudes (*r* = 0.369, *P* = 0.004).

#### SAI with paradoxical facilitation and presence of C-reflex in FAME2

Finally, we analysed the impact of altered excitability of M1 and S1 on sensorimotor integration by using the SAI paradigm. We found a *ISI* effect [*F*(4,248) = 35.480, *P* < 0.001], being the inhibition weaker at longer ISI and a *GROUP* effect [*F*(2,62) = 19.714, *P* < 0.001] with FAME2 patients exhibiting reduced SAI respect to JME (*P* < 0.001) and HC (*P* < 0.001). Interestingly the physiological sensory inhibition over M1 was replaced by paradoxical facilitation (Fig. 2H). No significant difference was observed between JME and HC (*P* = 0.738). The abnormal sensorimotor interaction, indexed by SAI, was also supported by the electrophysiological evidence of C-reflex in 80.77% of FAME2 patients (Supplementary Table 1). Individual SAI data are plotted in Fig. 3C and D.

Overall, these findings suggest an impairment of inhibitory cortical as well as subcortical circuits (i.e. thalamo-cortical connection). Likewise to M1 excitability, we explored the potential effect of benzodiazepines on S1 circuits, and no drug effect was evident on SEP components (unpaired *t*-test: N20, *P* = 0.538; P25, *P* = 0.345; N33, *P* = 0.785), paired-pulse SEP [*F*(1,23) = 0.442, *P* = 0.531], HFO [*F*(1,23) = 0.001, *P* = 0.994] and SAI [*F*(1,24) = 0.468, *P* = 0.500].

### Clinical correlates of neurophysiological abnormalities

Patients with a longer disease duration had a more severe myoclonus (*r* = 0.467, *P* = 0.016) associated to a lower frequency (*r* = −0.607, *P* = 0.001) and higher power of tremor (*r* = 0.479, *P* = 0.015). The peak frequency of the tremor was negatively associated with the peak power (*r* = −0.596, *P* = 0.002), meaning that the higher was the frequency and the lower was the tremor’s magnitude. No significant correlation was found between clinical parameters (age, disease duration, UMRS score, disease onset) and both TMS and SEP measures (all *P* > 0.05).

### Diagnostic accuracy of neurophysiological measures

A ROC curve was plotted to compare how effectively neurophysiologic measures differentiated FAME2 from JME and HC (Figs. 3A,B and 4A,B). Overall, TMS metrics showed higher diagnostic accuracy compared to SEP measures. Specifically, mean SICI showed an AUC of 0.97 (*P* < 0.0001), mean SAI of 0.85 (*P* = 0.0001), mean LICI of 0.99 (*P* < 0.0001) and RMT of 0.95 (*P* < 0.0001) (Fig. 3A). Regarding SEP measures, N20 amplitude displayed an AUC of 0.74 (*P* = 0.009), P25 of 0.68 (*P* = 0.049) and N33 of 0.89 (*P* < 0.0001) (Fig. 4A). Applying the best cut-off score for SICI of 66.6% and LICI of 35.02% yielded a sensitivity of 88.46% and 88.46%, specificity of 94.12% and 100%, the positive predictive value of 95.83% and 100%, the negative predictive value of 84.21% and 85%, and accuracy of 90.7% and 93.02%, respectively (Table 2). Among SEP measures, N33 amplitude displayed the highest accuracy of 81.4% applying the best cut-off score of 8.7 µV (sensitivity: 80.77%, specificity: 82.35%, positive predictive value: 87.5%, negative predictive value: 73.68%) (Table 2).

**Table 2:** AUC, sensitivity, specificity, positive and negative predictive values, and accuracy for receiver operating characteristic curves using best neurophysiologic parameters

Neurophysiologic measure, best cut-off	AUC (95% CI)	*P-*value	Sensitivity, %	Specificity, %	Positive predictive value, %	Negative predictive value, %	Accuracy, %
*FAME2 versus JME*							
SEP measures							
SEP N20 amplitude, <1.5 μV	0.74 (0.58–0.89)	0.009	65.38	70.59	77.27	57.14	67.44
SEP P25 amplitude, >7.3 μV	0.68 (0.51–0.85)	0.049	57.69	58.82	60.47	68.18	58.14
SEP N33 amplitude, >8.7 μV	0.89 (0.79–0.98)	<0.0001	80.77	82.35	87.5	73.68	81.4
e-HFO area, <1.6 µV × ms	0.82 (0.69–0.95)	0.002	76.92	75	86.96	60	76.32
l-HFO area, >2.4 µV × ms	0.79 (0.62–0.96)	0.004	73.08	75	86.36	56.25	73.68
TMS measures							
RMT%, <28.5%	0.95 (0.9–1)	<0.0001	80.77	100	100	77.27	88.37
AMT%, <24.5%	0.94 (0.87–1)	<0.0001	76.92	100	100	73.91	86.05
SICI%, >66.6%	0.97 (0.92–1)	<0.0001	88.46	94.12	95.83	84.21	90.7
LICI%, >35.02%	0.99 (0.97–1)	<0.0001	88.46	100	100	85	93.02
SAI%, >80.77%	0.85 (0.73–0.97)	0.0001	80.77	94.12	95.45	76.19	86.05
ICF%, <121.9%	0.66 (0.5–0.83)	0.074	61.54	64.71	72.73	52.38	62.79
SICF%, >159.8%	0.82(0.7–0.95)	0.0004	73.08	76.47	82.61	65	74.42
CSP (ms), <91.75 ms	0.91 (0.81–1)	<0.0001	80.95	93.75	94.44	78.95	86.49
*FAME2 versus HC*							
SEP measures							
SEP N20 amplitude, <2 μV	0.83 (0.7–0.95)	0.0001	80.77	81.82	84	78.26	81.25
SEP P25 amplitude, >6.8 μV	0.63 (0.47–0.79)	0.13	61.54	59.09	64	56.52	60.42
SEP N33 amplitude, >8.8 μV	0.87 (0.78–0.97)	<0.0001	76.92	81.82	83.33	75	79.17
e-HFO area, <1.8 µV × ms	0.83 (0.71–0.95)	0.0001	76.92	77.27	80	73.91	77.08
l-HFO area, >2.8 µV × ms	0.63 (0.47–0.79)	0.13	61.54	59.09	64	56.52	60.42
TMS measures							
RMT%, <28.5%	0.92 (0.85–1)	<0.0001	80.77	90.91	91.3	80	85.42
AMT%, <23.5%	0.82 (0.7–0.94)	0.0002	69.23	77.27	78.26	68	72.92
SICI%, >63.72%	0.97 (0.92–1)	<0.0001	92.31	90.91	92.31	90.91	91.67
LICI%, >47.91%	0.96 (0.91–1)	<0.0001	84.62	95.45	95.65	84	89.1
SAI%, >93.29%	0.87 (0.75–0.98)	<0.0001	80.77	90.91	91.3	80	85.42
ICF%, <119.6%	0.67 (0.52–0.83)	0.043	61.54	59.09	64	56.52	60.42
SICF%, >171.82%	0.79(0.68–0.92)	0.0004	69.23	72.73	75	66.67	70.83
CSP (ms), <91.83 ms	0.90 (0.8–1)	<0.0001	80.95	95.24	94.12	83.33	87.8

AUC = area under the curve; CI = confidence interval; FAME2 = familial adult myoclonic epilepsy type 2; HC = healthy controls; JME = juvenile myoclonic epilepsy; AMT = active motor threshold; RMT = resting motor threshold; SAI = mean short-latency afferent inhibition (0, 2, 4, 6, 8 ms); SICI = mean short-interval intracortical inhibition (ISI_peak1, ISI_trough, ISI_peak2), LICI = mean long-interval intracortical inhibition (100 and 150 ms); SICF = mean short-latency intracortical facilitation (1.0–3.6 ms with 0.2 ms step), CSP = mean cortical silent period (110%, 130%, 150% RMT); ICF = mean intracortical facilitation (10 and 15 ms); TMS = transcranial magnetic stimulation; SEP = somatosensory evoked potential; e-HFO = early high-frequency oscillations; l-HFO = late high-frequency oscillations.

Similar findings were observed when differentiating FAME2 from HC, with TMS measures being more accurate compared to SEP measures (Figs. 3B and 4B). Indeed, SICI showed an AUC of 0.97 (*P* < 0.0001), mean SAI of 0.87 (*P* < 0.0001), mean LICI of 0.96 (*P* < 0.0001) and RMT of 0.92 (*P* < 0.0001) (Fig. 3B), whereas N20 amplitude displayed an AUC of 0.83 (*P* = 0.0001), P25 of 0.63 (*P* = 0.13) and N33 of 0.87 (*P* < 0.0001) (Fig. 4B). Again, the highest accuracy was observed for the TMS metric, especially for SICI (91.67%) and LICI (89.1%), whereas SEP metrics displayed an overall lower accuracy (Table 2). For the comparison between JME and HC, see Supplementary Table 2.

Finally, the direct comparison of ROC curves, resulting from each TMS and SEP parameter in FAME2 and JME patients, disclosed that the AUC of LICI was significantly higher than the AUCs obtained for all SEP measures (all *P* < 0.04). Analogously, for the comparison between FAME2 and HC, we found that the AUC of SICI was significantly higher than the AUCs of all SEP metrics (all *P* < 0.04) (see Supplementary Table 3 for further details).

## Discussion

By using an extensive neurophysiological battery in our large sample of genetically confirmed FAME2 patients, we observed increased cortical facilitation and decreased inhibition in the sensorimotor cortex, associated with functional impairment of thalamo-cortical connection. Neither cortical nor thalamo-cortical circuit dysfunctions were associated with clinical severity. Noteworthy, TMS metrics, especially SICI and LICI, predicted FAME2 with much higher diagnostic usefulness than the conventional SEP parameters (i.e. P25 and N33 amplitudes).

Finally, patients with a longer disease duration had more severe myoclonus associated with a lower frequency and a higher tremor power, even if such progression was not associated with a concomitant increase of cortical hyperexcitability.

The latter finding is in line with our previous study^46^ showing a gradual and progressive worsening of the myoclonus overtime, which was correlated with the duration of the disease and with patients’ age. Interestingly, Neshige *et al.*^47^ suggested that at least two mechanisms might be involved in time-dependent neuronal dysfunction, such as repeat expansion causing cellular toxicity in the long-term and continuous excessive hyperexcitability state or bombardment of epileptic discharges.

### Cortical and subcortical contribution to the disrupted inhibitory/excitatory balance

The M1 of FAME2 patients is characterized by a significant reduction of γ-aminobutyric acid (GABA)-mediated inhibitory circuits, as probed by SICI, LICI and CSP.^48^ These data are in line with previous reports on very small samples of patients, showing reduced SICI^10,11^ and shorter CSP.^9,10^ In our large series of patients, we consistently demonstrated impairment of both GABA-A, indexed by SICI, and GABA-B circuits, indexed by LICI and CSP, and more interestingly such alterations were present early in the course of the disease and were not influenced by disease progression and benzodiazepine therapy.

We also demonstrated a marked increase of facilitatory circuits, probed by motor thresholds and SICF. Both paradigms seem to be partly underpinned by glutamatergic *N*-methyl-d-aspartate (NMDA)-type and α-amino-3-hydroxy-5-methyl-4-isoxazolepropionic acid (AMPA)-type receptors (Table 1) other than GABA-A circuits for SICF (Table 1). Previous literature on motor thresholds gave conflicting findings, with one study describing normal findings^11^ and another one with reduced motor thresholds.^10^ Overall, our results support the concept that FAME2 exhibits higher corticospinal excitability compared with HC and JME. On the contrary, higher cortical thresholds in JME might be explained by the greater percentage of patients taking lamotrigine, a sodium channel blocker with a well-known effect on increasing motor thresholds.^25,48^ In addition, the normal findings of GABA-B circuits in JME are in line with previous studies.^20,49^ As for SICI, we found a normal inhibitory profile in JME patients; this result fits well with a previous study demonstrating the normalization of inhibitory circuits at short latency in controlled JME compared to chronic refractory JME.^50^ Finally, none of the M1 metrics correlated with clinical features, including disease duration, myoclonus severity and disease onset.

Furthermore, FAME2 patients exhibited a prominent alteration of S1 inhibitory circuits indexed by a significant increase of the paired-pulse ratio at a short interval (i.e. ISI of 5 ms). The reduction of cortical inhibition could also explain the giant SEP and a larger area of l-HFO that we observed in FAME2 patients. Indeed, l-HFO are probably originated from the activity of inhibitory interneuron that produces feedforward inhibition of pyramidal neuron within S1,^31^ and previous studies have reported larger amplitudes of l-HFO in patients with myoclonic epilepsy,^51^ non-progressive myoclonic epilepsy,^52^ parkinsonism with myoclonus^52^ and very recently also in Japanese patients with benign adult familial myoclonus epilepsy.^53^

Despite the prominent S1 hyperexcitability observed in FAME2, none of the neurophysiological parameters was significantly associated with disease severity and age.

This finding seems to be in contrast with two previous cross-sectional studies that suggest a significant relationship between giant SEP and myoclonus severity^54^ and aging.^47^ A possible explanation may be the higher amount of ASMs that could reduce somatosensory cortex excitability in patients with more severe disease or possible concomitant cortical atrophy mechanisms in the later disease stage. However, only future longitudinal studies could address this important question.

Besides cortical hyperexcitability, the lack of suppression at longer ISIs (i.e. 20 and 40 ms) during the SEP recovery cycle suggests the concomitant involvement of subcortical networks, possibly involving dorsal column nuclei and the thalamus (ventral postero-lateral nucleus).^37,38^ This aspect is also corroborated by the significant reduction of the e-HFO that are considered presynaptic spikes produced by thalamo-cortical axon terminals at the time when they arrive at the somatosensory cortex.^31^

The involvement of subcortical circuits is of interest since ‘rhythmicity' of cortical myoclonic tremor could derive from an unstable loop between cortex and subcortical structures.^5^ Interestingly, the relay nuclei of the thalamus can demonstrate oscillatory behaviour due to a combination of intrinsic properties of ion channels in individual neurons and because of the way the neurons of the thalamus are interconnected within the central nervous system circuits.^55^

Therefore, jerk rhythmicity in cortical tremors could be explained by the interaction between local excitatory–inhibitory circuits within the sensorimotor cortex and synchronization with external sources such as the thalamus. When this thalamo-cortical sensorimotor circuit becomes disrupted cortical tremor might appear.

We have also demonstrated that sensorimotor integration was deeply altered in FAME2 patients, and more interestingly the physiological sensory inhibition over the motor cortex^56^ was replaced by facilitation. The paradoxical SAI facilitation is in agreement with previous literature of patients with progressive myoclonic epilepsy^57,58^

Regarding JME, we observed normal SAI, as previously described,^49^ also HFO areas did not significantly differ from HC, suggesting the role of ASMs in reducing thalamic^22^ and cortical hyperexcitability in these patients.^59^

### Measures relative to M1 have better diagnostic accuracy than those related to S1

The systematic evaluation of diagnostic accuracy of neurophysiological measures suggests that the conventional SEP has an overall low diagnostic accuracy and low negative predicting value, meaning that the absence of giant SEP does not exclude at all the diagnosis. This finding is not surprising if we look at the presence of giant SEP occurring in previous cohorts, ranging from 0% reported by Suppa *et al*.^10^ to 77.6% in a Japanese cohort^60^ and 87.5% described by Cen *et al*.^54^ In line with this heterogeneous data, our study confirms that conventional SEP might not be accurate enough for diagnosis especially in patients with mild phenotype or at the beginning of the disease. We also observed a low diagnostic accuracy for l-HFO, this finding seems to be in contrast with a recent study that showed the presence of l-HFO in Benign adult familial myoclonic epilepsy (BAFME) but not in patients with cortical myoclonus caused by different conditions.^53^ However, in the Japanese study,^53^ the lack of an HC population does not allow to define l-HFO as specific for BAFME condition, since we herein demonstrated that l-HFO were recordable in HCs and JME too.

Finally, we demonstrated the higher diagnostic accuracy of TMS measures, especially SICI and LICI, compared with SEP parameters for predicting FAME2. This result might support the implementation of TMS in the diagnostic work up before genetic testing.

## Conclusion and outlook

Our study has some implications from a clinical point of view. First, the FAME2 condition can be considered as a disease model of aberrant sensory–motor excitability. Therefore, we suggest that implementing the systematic evaluation of cortical and subcortical circuits by using *ad hoc* TMS and SEP protocols might be of interest in the future investigation of the neural mechanisms underlying hyperexcitability of S1 and M1 in patients with amyotrophic lateral sclerosis.^61^ Analogously, the application of these neurophysiological procedures in stroke patients might uncover the mechanisms of increased cortical excitability that are essential to boost synaptic plasticity and promote the reorganization of cortical networks in the recovery phase.^62^

Second, the exaggerated cortical facilitation likely due to NMDA-type and/or AMPA-type glutamate transmission may provide a novel avenue for treatment targeting these specific receptors. Interestingly, anti-glutamatergic drugs such as perampanel, an AMPA-receptor antagonist, could be eventually helpful in this condition. Indeed, recent TMS^63^ and SEP studies^64^ have demonstrated its effect in increasing motor thresholds in the HC and reducing SEP amplitude and HFOs area in the JME, thus raising the possibility of normalizing excitatory circuits in the sensorimotor cortex with possible beneficial effects on cortical tremor in FAME2 patients.

## Supplementary Material

fcac037_Supplementary_Data

## Abbreviations

AMT =: active motor threshold
CSP =: cortical silent period
FAME2 =: familial adult myoclonic epilepsy type 2
HFOs =: high-frequency oscillations
ICF =: intracortical facilitation
JLA =: jerk-locked back averaging
JME =: juvenile myoclonic epilepsy
LICI =: long-interval intracortical inhibition
RMT =: resting motor threshold
SAI =: short-latency afferent inhibition
SEP =: somatosensory evoked potential
SICF =: short-interval intracortical facilitation
SICI =: short-interval intracortical inhibition
*STARD7* =: StAR-related lipid transfer domain-containing 7
TMS =: transcranial magnetic stimulation
UMRS =: Unified Myoclonus Rating Scale
