# Supplementary material for: Abnormal sensorimotor cortex and thalamo-cortical networks in familial adult myoclonic epilepsy type 2: pathophysiology and diagnostic implications
Source: Brain Commun. 2022 Feb 15;4(1):fcac037. doi: 10.1093/braincomms/fcac037 (PMC8882005; doi:10.1093/braincomms/fcac037)
Supplement: fcac037_Supplementary_Data [file fcac037_supplementary_data.pdf]

Supplementary Table 1

Clinical, demographic and neurophysiological findings in FAME2 and JME patients

Participant	Sex/Age (y)	Disease duration (y)	Current AEDs	UMRS (section 4)	EPILEPSY			CORTICAL TREMOR				SEP-amplitude (μV)			C-reflex	CI C-reflex
					Age at onset (y)	Type of seizures	Interictal EEG abnormalities	Age at onset (y)	Tremor frequency (Hz)	Tremor power ($\mu V^2/Hz$)	JLA (YES/NO)	N20	P25	N33	ABP (Latency)	ABP (Latency)
FAME2	F/60	42	LEV; CLN	25	44	GTCS	bifrontal sharp waves	18	8	232.2	YES	1.25	9.34	17.27	42.8	49.2
FAME2	M/61	29	LEV; VPA, CLB	16	32	GTCS	diffuse sharp waves	32	6.75	755	YES	1.43	11.01	14.78	45.6	-
FAME2	M/32	19	LEV; CLN	13	-	-	-	13	11.25	10.16	YES	4.72	18.76	16.55	46.2	-
FAME2	M/29	15	LEV	14	28	GTCS	diffuse sharp waves	14	7.4	66.3	YES	1.49	9.79	12.27	37.2	-
FAME2	F/52	32	LEV; VPA; CLN	28	20	myoclonic	diffuse sharp waves	20	6	370	YES	0.55	5.19	6.75	40.3	-
FAME2	F/45	19	LEV	9	-	-	left temporal spikes	26	9	42.3	YES	0.94	12.40	19.88	45	53
FAME2	M/43	20	LEV; VPA; CLN	17	36	GTCS	-	23	7.25	236	YES	4.68	21.73	35.07	48.4	-
FAME2	M/18	4	-	7	-	-	-	14	12	11.8	NA	4.06	19.36	22.22	-	-
FAME2	M/64	44	LEV; VPA; CLN	32	34	GTCS	-	20	6	460	YES	0.43	5.64	10.63	45.6	-
FAME2	M/39	16	LEV	11	24	GTCS	-	23	NA	NA	NA	0.53	6.00	9.00	42.2	-
FAME2	F/60	49	LEV; VPA	12	34	myoclonic	diffuse sharp waves	11	7.7	202.5	YES	1.84	19.31	33.26	41.3	-
FAME2	M/62	15	LEV; CLN	20	34	GTCS	bilateral frontal sharp waves	22	8.25	155.1	YES	2.01	13.64	19.21	-	-
FAME2	M/27	9	-	7	-	-	-	18	7.9	244	YES	1.94	4.85	5.96	-	-
FAME2	M/37	42	LEV	10	-	-	-	18	6	675	YES	0.35	3.02	4.99	42.8	50.5

FAME2	M/71	9	LEV; VPA	26	18	GTCS	fronto-temporo-occipital spikes	18	8.5	55.96	YES	0.80	7.28	12.44	43.9	52.7
FAME2	F/45	25	LTG	17	43	GTCS	NA	20	8.8	576	NA	0.70	3.56	8.46	45.5	-
FAME2	M/46	24	-	18	42	myoclonic	NA	22	9.6	250	NA	0.77	4.66	9.56	43.7	53.8
FAME2	M/55	42	VPA; CLB	25	25	GTCS	diffuse sharp waves	13	6.3	214	YES	1.08	2.80	9.40	43.4	-
FAME2	M/57	43	VPA	12	22	GTCS	bilateral fronto-temporal sharp waves	14	9.25	62.8	YES	1.80	8.40	13.10	42.8	-
FAME2	F/46	16	-	8	-	-	temporal spike-waves	30	10.5	10.7	NA	0.79	11.50	13.20	40.3	-
FAME2	F/51	13	VPA	10	38	GTCS	temporal spike-waves	38	8	231	YES	0.94	22.00	30.20	42.8	51.3
FAME2	M/60	32	LEV; VPA; PHB	24	28	GTCS	-	28	5.5	315	NA	1.01	8.7	13.1	-	-
FAME2	F/15	3	-	14	-	-	diffuse sharp waves	12	9.75	12.7	YES	8.00	20.00	14.50	39.7	-
FAME2	M/81	45	VPA; PHB	25	36	GTCS	bilateral fronto-temporal sharp waves	36	5	294.3	NA	1.16	4.74	3.87	48	-
FAME2	M/50	35	LTG; LEV; PHT; PHB	21	15	myoclonic	temporal spike-waves	15	6.5	196	YES	0.59	2.83	2.69	-	-
FAME2	M/32	8	CLN	14	-	-	-	24	8.5	54.3	NA	2.40	12.20	20.40	42.8	-
JME	M/40	21	VPA; LTG		19							1.78	5.15	1.2	-	-
JME	F/25	10	VPA; LTG		15							1.5	9.67	10.7	-	-
JME	M/46	27	VPA		19							3.96	10.4	7.35	-	-
JME	F/18	3	LTG		15							3.4	8.5	9.5	-	-
JME	F/52	31	VPA; LEV		21							1.55	2.69	1.49	-	-
JME	M/32	13	VPA; LTG		19							3.23	5.52	1.43	-	-
JME	F/21	6	LEV; LTG		15							1.99	3.29	2.75	-	-

JME	F/33	13	VPA; LTG		20							11	21.9	1.6	-	-
JME	F/55	47	VPA; LEV; PHB		8							1.52	2.27	1.47	-	-
JME	F/32	13	LEV		19							0.81	3	3.22	-	-
JME	F/58	37	LEV		21							1	1.55	0.432	-	-
JME	F/28	13	LEV		15							2.5	5.88	3.56	-	-
JME	M/33	15	LTG; LEV		18							2.9	4.2	2.9	-	-
JME	M/33	9	LTG; VPA; LEV		24							1.18	2.7	5.34	-	-
JME	M/42	8	LEV; VPA		34							3.93	7.97	4	-	-
JME	F/27	13	LEV		14							5.5	18.8	14.4	-	-
JME	M/42	26	LEV; VPA		16							3.24	7.37	4.5	-	-

Abbreviations: -= absent; C= cortical; Cl= contralateral; CLB= clobazam; CLN = clonazepam; FAME2= familial adult myoclonic epilepsy type 2; GTCS= generalized tonic clonic seizure; JLA= jerk-locked back averaging; JME= juvenile myoclonic epilepsy; LEV= levetiracetam; LTG = lamotrigine; NA= not available; PHB= phenobarbital; PHT= phenytoin; SEP= somatosensory evoked potential; UMRS= Unified Myoclonus Rating Scale; VPA= valproate. For UMRS (section 4), higher scores indicate more severe involuntary movements.

Supplementary Table 2

AUC, sensitivity, specificity, positive and negative predictive values, and accuracy for receiver operating characteristic curves using best neurophysiologic parameters

Neurophysiologic measure, best cut-off	AUC (95% CI)	p value	Sensitivity, %	Specificity, %	Positive predictive value, %	Negative predictive value, %	Accuracy, %
**HC vs JME**							
**SEP measures**							
SEP N20 amplitude, <2.5 µV	0.64 (0.46-0.820)	0.14	52.94	63.64	52.94	63.64	58.97
SEP P25 amplitude, <3.7 µV	0.57 (0.38-0.77)	0.43	41.18	68.18	50	60	56.41
SEP N33 amplitude, <4.3 µV	0.52 (0.34-0.71)	0.83	64.71	40.91	45.83	60	51.28
e-HFO area, <2.15 µV x ms	0.51 (0.28-0.73)	0.96	50	54.55	37.5	66.67	52.94
l-HFO area, <2.1 µV x ms	0.69 (0.5-0.88)	0.063	66.67	63.64	50	77.78	64.71
**TMS measures**							
RMT%, >39.5%	0.67 (0.5-0.86)	0.058	58.82	68.18	58.82	68.18	64.1
AMT%, > 31.5%	0.74 (0.58-0.89)	0.01	52.94	81.82	69.23	69.23	69.23
SICI%, >42.04%	0.54 (0.36-0.73)	0.65	41.18	59.09	43.75	56.52	51.28
LICI%, <15.55%	0.68 (0.51-0.85)	0.054	76.47	50	54.17	73.33	61.54
SAI%, >79.19%	0.61 (0.43-0.79)	0.26	70.59	45.45	50	66.67	56.41
ICF%, <126.1%	0.51 (0.31-0.69)	0.95	47.06	54.55	44.44	57.14	51.28
SICF%, <169.2%	0.53 (0.35-0.72)	0.73	64.71	50	50	64.71	56.41
CSP(ms), >120.5 ms	0.62 (0.43-0.82)	0.19	68.75	57.14	55	70.59	62.16

Abbreviations: AUC= area under the curve; CI = confidence interval; HC = healthy controls; JME= juvenile myoclonic epilepsy; AMT= active motor threshold; RMT= resting motor threshold; SAI= mean short-latency afferent inhibition (0, 2, 4, 6, 8 milliseconds); SICI= mean short-interval intracortical inhibition (ISI_peak1, ISI_trough, ISI_peak2), LICI= mean long-interval intracortical inhibition (100 and 150 milliseconds); SICF= mean short-latency intracortical facilitation (1.0 to 3.6 ms with 0.2 ms step), CSP= mean cortical silent period (110%, 130%, 150% RMT); ICF= mean intracortical facilitation (10 and 15 milliseconds); TMS= transcranial magnetic stimulation; SEP= somatosensory evoked potential; e-HFO= early high frequency oscillations; l-HFO= late high frequency oscillations.

Supplementary Table 3

Direct comparison between the AUC of TMS measures vs SEP parameters

**FAME2 vs JME**		**RMT%**	**AMT%**	**SICI%**	**LICI%**	**SAI%**	**ICF%**	**SICF%**	**CSP (ms)**
**SEP N20 amplitude**	**AUC difference ± SE**	0.21 ± 0.08	0.19 ± 0.07	0.23 ± 0.08	0.23 ± 0.08	0.09 ± 0.09	0.08 ± 0.11	0.08 ± 0.09	0.16 ± 0.09
	**p value**	**0.005**	**0.01**	**0.003**	**0.003**	0.32	0.5	0.4	0.08
**SEP P25 amplitude**	**AUC difference ± SE**	0.27 ± 0.08	0.26 ± 0.08	0.29 ± 0.08	0.32 ± 0.09	0.19 ± 0.1	0.016 ± 0.12	0.15 ± 0.1	0.25 ± 0.09
	**p value**	**0.001**	**0.002**	**0.0006**	**0.0004**	0.07	0.9	0.15	**0.01**
**SEP N33 amplitude**	**AUC difference ± SE**	0.06 ± 0.05	0.05 ± 0.05	0.08 ± 0.05	0.1 ± 0.03	0.02 ± 0.08	0.23 ± 0.09	0.08 ± 0.09	0.04 ± 0.07
	**p value**	0.2	0.4	0.14	**0.04**	0.8	**0.015**	0.4	0.6
**e-HFO area**	**AUC difference ± SE**	0.12 ± 0.07	0.13 ± 0.07	0.14 ± 0.07	0.18 ± 0.07	0.002 ± 0.09	0.04 ± 0.1	0.07 ± 0.07	0.07 ± 0.08
	**p value**	0.08	0.18	0.05	**0.008**	1	0.7	0.4	0.4
**l-HFO area**	**AUC difference ± SE**	0.15 ± 0.09	0.12 ± 0.09	0.16 ± 0.08	0.21 ± 0.08	0.08 ± 0.11	0.15 ± 0.13	0.15 ± 0.12	0.15 ± 0.1
	**p value**	0.08	0.18	0.05	**0.02**	0.5	0.3	0.2	0.14

**FAME2 vs HC**		**RMT%**	**AMT%**	**SICI%**	**LICI%**	**SAI%**	**ICF%**	**SICF%**	**CSP (ms)**
**SEP N20 amplitude**	**AUC difference ± SE**	0.09 ± 0.07	0.01 ± 0.08	0.14 ± 0.07	0.09 ± 0.07	0.01 ± 0.08	0.04 ± 0.11	0.17 ± 0.1	0.04 ± 0.08
	**p value**	0.2	0.9	**0.04**	0.2	0.9	0.7	0.07	0.6
**SEP P25 amplitude**	**AUC difference ± SE**	0.3 ± 0.08	0.2 ± 0.1	0.34 ± 0.08	0.36 ± 0.09	0.3 ± 0.09	0.04 ± 0.11	0.17 ± 0.1	0.3 ± 0.1
	**p value**	**0.0004**	0.05	**<0.0001**	**0.0001**	**0.004**	0.7	0.07	**0.001**
**SEP N33 amplitude**	**AUC difference ± SE**	0.05 ± 0.06	0.06 ± 0.08	0.1 ± 0.03	0.09 ± 0.06	0.01 ± 0.07	0.2 ± 0.09	0.08 ± 0.08	0.04 ± 0.07
	**p value**	0.4	0.4	**0.04**	0.1	0.8	**0.02**	0.3	0.6
**e-HFO area**	**AUC difference ± SE**	0.1 ± 0.07	0.01 ± 0.08	0.13 ± 0.07	0.13 ± 0.06	0.03 ± 0.08	0.2 ± 0.1	0.002 ± 0.1	0.05 ± 0.08
	**p value**	0.2	0.9	**0.03**	**0.03**	0.7	0.08	1	0.6
**l-HFO area**	**AUC difference ± SE**	0.3 ± 0.09	0.19 ± 0.1	0.34 ± 0.08	0.33 ± 0.08	0.3 ± 0.1	0.16 ± 0.11	0.03 ± 0.09	0.3 ± 0.09
	**p value**	**0.0007**	0.05	**<0.0001**	**<0.0001**	**0.005**	0.2	0.8	**0.0013**

JME vs HC		RMT%	AMT%	SICI%	LICI%	SAI%	ICF%	SICF%	CSP (ms)
**SEP N20 amplitude**	**AUC difference ± SE**	0.08 ± 0.14	0.13 ± 0.13	0.08 ± 0.14	0.21 ± 0.12	0.04 ± 0.13	0.01 ± 0.14	0.009 ± 0.15	0.17 ± 0.15
	**p value**	0.55	0.34	0.55	0.09	0.74	0.9	0.9	0.25
**SEP P25 amplitude**	**AUC difference ± SE**	0.05 ± 0.13	0.09 ± 0.13	0.04 ± 0.14	0.17 ± 0.12	0.004 ± 0.15	0.03 ± 0.15	0.04 ± 0.15	0.14 ± 0.14
	**p value**	0.7	0.5	0.8	0.16	1	0.9	0.8	0.34
**SEP N33 amplitude**	**AUC difference ± SE**	0.05 ± 0.12	0.09 ± 0.12	0.04 ± 0.13	0.17 ± 0.13	0.008 ± 0.13	0.02 ± 0.14	0.04 ± 0.14	0.14 ± 0.13
	**p value**	0.7	0.4	0.7	0.18	1	0.9	0.8	0.31
**e-HFO area**	**AUC difference ± SE**	0.11 ± 0.14	0.15 ± 0.13	0.11 ± 0.14	0.24 ± 0.11	0.07 ± 0.15	0.04 ± 0.14	0.009 ± 0.15	0.17 ± 0.14
	**p value**	0.4	0.3	0.4	**0.04**	0.6	0.8	1	0.23
**l-HFO area**	**AUC difference ± SE**	0.08 ± 0.13	0.03 ± 0.11	0.15 ± 0.12	0.05 ± 0.12	0.15 ± 0.13	0.2 ± 0.15	0.2 ± 0.13	0.002 ± 0.14
	**p value**	0.54	0.77	0.22	0.7	0.26	0.17	0.16	1

Abbreviations: AUC= area under the curve; FAME2= Familial Adult Myoclonic Epilepsy type2; HC = healthy controls; JME= juvenile myoclonic epilepsy; AMT= active motor threshold; RMT= resting motor threshold; SAI= mean short-latency afferent inhibition (0, 2, 4, 6, 8 milliseconds); SICI= mean short-interval intracortical inhibition (ISI_peak1, ISI_trough, ISI_peak2), LICI= mean long-interval intracortical inhibition (100 and 150 milliseconds); SICF= mean short-latency intracortical facilitation (1.0 to 3.6 ms with 0.2 ms step), CSP= mean cortical silent period (110%, 130%, 150% RMT); ICF= mean intracortical facilitation (10 and 15 milliseconds); TMS= transcranial magnetic stimulation; SE= standard error; SEP= somatosensory evoked potential; e-HFO= early high frequency oscillations; l-HFO= late high frequency oscillations. In bold significant $p < 0.05$.

Supplementary data

1. **Transcranial magnetic stimulation protocols**

TMS was performed using a figure-of-8–shaped magnetic coil (MC-B70 Butterfly: outer diameter of each wing 97 mm) that was held tangentially to the skull with the handle pointing backwards and laterally at an angle of 45° to the sagittal plane (direction of current induced in the brain: posterior to anterior).

The "hot spot" was defined as the scalp position over the left primary motor area where maximal motor evoked potentials (MEPs) was elicited in the contralateral first dorsal interosseous (FDI) muscle (Groppa *et al.*, 2012). To ensure the stability of the stimulation position over the course of the experiment, the hotspot was marked directly on the scalp with a soft-tip pen. The signal was acquired via Ag–AgCl surface electrodes in a belly tendon montage, amplified, bandpass filtered (20 Hz-3 kHz) and digitized at a frequency of 5 kHz (Signal software and CED 1401 hardware, Cambridge Electronic Design, Cambridge, UK).

Resting motor threshold (RMT), given in percentage of maximum stimulator output, was defined as the minimum stimulus intensity that produced at rest a MEP of 50 μV in at least 5 of 10 trials (Rossini *et al.*, 2015). Active motor threshold (AMT) was calculated during a mild tonic contraction (approximately 20% of maximal contraction) as the lowest intensity evoking five MEPs of at least 200 μV in ten consecutive trials (Rossini *et al.*, 2015). Visual feedback was provided through an oscilloscope placed in front of the subject to ensure a constant force level.

Finally, MEP1mV was determined as the stimulus intensity, which elicited in the resting FDI a MEP of 1 mV on average.

Short-interval intracortical facilitation (SICF) was evaluated as a function of fifteen inter-stimulus intervals (ISIs, Fig 1B), ranging from 1.0 to 3.8 ms with 0.2 ms step, between the first stimulus set to MEP1mV and the second stimulus at 90% RMT (Ziemann *et al.*, 1998; Hanajima *et al.*, 2002).

Short-interval intracortical inhibition (SICI) and intracortical facilitation (ICF) were determined by setting the conditioning stimulus (CS) intensity to 95% AMT and delivering the CS before the test stimulus (TS) set to 1 mV. The ISIs used for SICI were modelled on the individual first peak (*peak1*), first trough (*trough*) and second peak (*peak2*) obtained from the SICF curve (Peurala *et al.*, 2008; Ni *et al.*, 2013), while intracortical ICF was determined at ISIs of 10 and 15 ms. Long-interval intracortical inhibition (LICI) was investigated by implementing 2 suprathreshold stimuli (MEP1mV) with ISIs of 100 and 150 milliseconds (Nakamura *et al.*, 1997).

The mean length of the cortical silent period (CSP) was measured from the onset of the motor potential to the resumption (at any level) of sustained EMG activity. The CSP was evoked with single-pulse TMS during continuous isometric contraction of the target muscle. Stimulus intensity was set

at 110, 120 and 130% of RMT and 15 traces were recorded for each stimulation intensity. Subjects were asked to produce an isometric contraction at ~50% of maximal voluntary contraction. Visual feedback was provided through an oscilloscope placed in front of the subject to ensure a constant force level.

For all paired pulse protocols, the unconditioned MEP (TS) was adjusted to evoke a MEP of 1 mV amplitude in the right FDI muscle, 15 trials were recorded for each condition and randomly intermixed with 15 trials of TS alone (0.2 Hz ± 10%). The ratio of the mean amplitude of the conditioned response to that of the TS response was calculated for each condition and ISI in each subject.

References

Groppa S, Oliviero A, Eisen A, Quartarone A, Cohen LG, Mall V, et al. A practical guide to diagnostic transcranial magnetic stimulation: Report of an IFCN committee. Clin Neurophysiol 2012; 123: 858–882.

Hanajima R, Ugawa Y, Terao Y, Enomoto H, Shiio Y, Mochizuki H, et al. Mechanisms of intracortical I-wave facilitation elicited with paired-pulse magnetic stimulation in humans. J Physiol 2002; 538: 253–261.

Nakamura H, Kitagawa H, Kawaguchi Y, Tsuji H. Intracortical facilitation and inhibition after transcranial magnetic stimulation in conscious humans. J Physiol 1997; 498 (Pt 3: 817–823.

Ni Z, Bahl N, Gunraj CA, Mazzella F, Chen R. Increased motor cortical facilitation and decreased inhibition in Parkinson disease. Neurology 2013; 80: 1746–1753.

Peurala SH, M. Müller-Dahlhaus JF, Arai N, Ziemann U. Interference of short-interval intracortical inhibition (SICI) and short-interval intracortical facilitation (SICF). Clin Neurophysiol 2008; 119: 2291–2297.

Rossini PM, Burke D, Chen R, Cohen LG, Daskalakis Z, Di Iorio R, et al. Non-invasive electrical and magnetic stimulation of the brain, spinal cord, roots and peripheral nerves: Basic principles and procedures for routine clinical and research application: An updated report from an I.F.C.N. Committee. Clin Neurophysiol 2015; 126: 1071–1107.

Ziemann U, Tergau F, Wassermann EM, Wischer S, Hildebrandt J, Paulus W. Demonstration of facilitatory I wave interaction in the human motor cortex by paired transcranial magnetic stimulation. J Physiol 1998; 511: 181–190.

Supplementary Figure 1

Antiseizure Medications (ASMs) in FAME2 vs JME

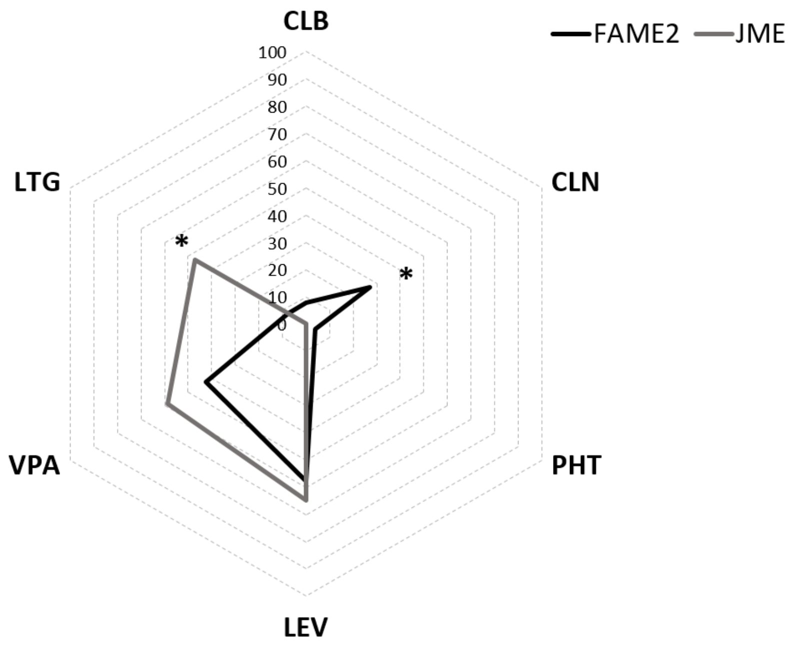

Antiseizures medications (ASMs) in FAME2 and JME patients

Spider plot showing the percentage of patients (FAME2: black line and JME: grey line) under each ASM. *= statistically significant difference computed with Chi-square test.

Abbreviations: CLB= clobazam; CLN = clonazepam; FAME2= familial adult myoclonic epilepsy type 2; JME= juvenile myoclonic epilepsy; LEV= levetiracetam; LTG= lamotrigine; PHB= phenobarbital; PHT= phenytoin; VPA= valproate.
